# Deciphering the pathogenesis of the COL4‐related hematuric nephritis: A genotype/phenotype study

**DOI:** 10.1002/mgg3.1576

**Published:** 2020-12-24

**Authors:** Vera Uliana, Paola Sebastio, Matteo Riva, Diana Carli, Claudio Ruberto, Laura Bianchi, Claudio Graziano, Irene Capelli, Flavio Faletra, Roberto Pillon, Teresa Mattina, Alberto Sensi, Francesco Bonatti, Antonio Percesepe

**Affiliations:** ^1^ Medical Genetics University Hospital of Parma Parma Italy; ^2^ Medical Genetics Department of Medicine and Surgery University of Parma Parma Italy; ^3^ Medical Genetics University Hospital “Città della Salute” Torino Italy; ^4^ Pediatrics University Hospital of Parma Parma Italy; ^5^ Medical Genetics S. Orsola‐Malpighi University Hospital Bologna Italy; ^6^ Nephrology S. Orsola‐Malpighi University Hospital Bologna Italy; ^7^ Medical Genetics I.R.C.C.S. Burlo Garofolo Trieste Italy; ^8^ Medical Genetics University of Trieste Trieste Italy; ^9^ Medical Genetics Centro di Riferimento Regionale per la Diagnosi e Cura della Malattie Genetiche Catania Italy; ^10^ Medical Genetics Maurizio Bufalini Hospital Cesena Italy

**Keywords:** Alport syndrome, *COL4A3*, *COL4A4* gene mutations, *COL4A5*, genotype/phenotype

## Abstract

**Background:**

Alport syndrome (ATS) is a hereditary progressive hematuric nephropathy associated with sensorineural deafness and ocular abnormalities, which is caused by mutations in the *COL4A5* gene (X‐linked ATS) and in two autosomal genes, *COL4A4* and *COL4A3*, responsible of both recessive ATS and, when present in heterozygosity, of a spectrum of phenotypes ranging from isolated hematuria to frank renal disease.

**Methods:**

Retrospective analysis of the clinical and genetic features of 76 patients from 34 unrelated ATS families (11 with mutations in *COL4A5*, 11 in *COL4A3*, and 12 in *COL4A4*) and genotype/phenotype correlation for the *COL4A3*/*COL4A4* heterozygotes (34 patients from 14 families).

**Results:**

Eight (24%) of the 34 heterozygous *COL4A3* and *COL4A4* carriers developed renal failure at a mean age of 57 years, with a significantly lower risk than hemizygous *COL4A5* or double heterozygous *COL4A3*/*COL4A4* carriers (*p* < 0.01), but not different from that of the heterozygous *COL4A5* females (*p* = 0.6). Heterozygous carriers of frameshift/splicing variants in *COL4A3*/*COL4A4* presented a higher risk of developing renal failure than those with missense variants in the glycine domains (*p* = 0.015).

**Conclusion:**

The renal functional prognosis of patients with *COL4A3*/*COL4A4*‐positive ATS recapitulates that of the X‐linked ATS forms, with differences between heterozygous vs. double heterozygous patients and between carriers of loss‐of‐function vs. missense variants.

## Graphical abstract

Heterozygotes for COL4A3 and COL4A4 variants showed a significantly lower risk than hemizygous COL4A5 or double heterozygous COL4A3/COL4A4 carriers (*p* < 0.01), but not different from that of the heterozygous COL4A5 females (*p* = 0.6). Heterozygote carriers of frameshift/splicing variants in COL4A3/COL4A4 presented a higher risk of developing renal failure than those with missense variants in the glycine domains (*p* = 0.015).

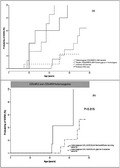

## 1 INTRODUCTION

Alport syndrome (ATS) is a heterogeneous group of progressive nephropathies featured by the variable association of hematuric nephritis with ultrastructural changes of the glomerular basement membrane (thinning, thickening, and splitting), sensorineural deafness, and ocular abnormalities (anterior lenticonus, macular flecks, and cataract) (Kashtan & Michael, 1996). The disease is characterized by the alteration of the type IV collagen network of the glomerular basement membrane (GBM), which is due to mutations in one gene mapping on the X chromosome, *COL4A5* (OMIM*303630), and two genes on the autosomes, *COL4A3* (OMIM*120070) and *COL4A4* (OMIM*120131), thus encompassing a genetic pattern of transmission including X‐linked, autosomal and digenic inheritance (Kashtan & Michael, 1996; Savige et al., 2013). By leading 70% of the affected males to end‐stage renal disease (ESRD) before the age of 30 years (Jais et al., 2000, 2003), the X‐linked ATS (XLS, OMIM #301050) is burdened by a severe prognosis, as well as the autosomal recessive form (ATS 2, OMIM #203780), which is associated with the early onset of a ESRD in both males and females (Lee et al., 2019). On the other hand, the female carriers of the XLS and the carriers of heterozygous *COL4A4* and *COL4A3* mutations have a better prognosis, featured by a spectrum of phenotypes ranging from a complete absence of signs and symptoms of kidney disease to isolated hematuria, up to progressive renal disease arising in a few cases (Jais et al., 2003; Matthaiou et al., 2020). There is a lack of consensus on the definition of this latter condition, which has been variously classified as thin basement membrane nephropathy or autosomal dominant Alport syndrome (ATS 3, OMIM #104200) (Fallerini et al., 2014; Savige, 2018).

ATS has been underdiagnosed for several decades for the broad phenotypic spectrum associated with abnormalities of the collagen IV α345 molecules and for the intrinsic difficulties of the molecular testing, due to the genetic heterogeneity of the disease, to the large size of the three genes involved and the lack of mutational hot spots (Jais et al., 2000, 2003). The recent widespread adoption of NGS technologies by routine diagnostic laboratories has allowed an easier access to a comprehensive genetic testing for ATS and has finally improved the diagnosis and research for the disease (Fallerini et al., 2014; Morinière et al., 2014). The present study reports the clinical and genetic features of 76 patients from 34 unrelated ATS families with mutations in *COL4A3*, *COL4A4*, or *COL4A5* variants identified using NGS technology, with a special focus on the genotype/phenotype correlations in patients with heterozygous *COL4A3* and *COL4A4* variants.

## 2 PATIENTS AND METHODS

The analysis is based on the records of the Unit of Medical Genetics of the Parma University Hospital and covers the period June 2016‐November 2019 reporting the results of the genetic testing performed by next‐generation sequencing using the Illumina MiSeq platform (TruSeq Custom Amplicon v.1.5), with a gene panel including *COL4A5* (GenBank reference sequence, RefSeq: NM_033380.3), *COL4A3* (RefSeq: NM_000091.5), and *COL4A4* (RefSeq: NM_000092.5) on patients with hematuria or chronic renal failure also meeting at least one of the four clinical criteria for ATS: a positive family history of macro/microscopic hematuria or ESRD; electron microscopic evidence of ATS on renal biopsy; characteristic ophthalmic signs; high‐tone sensorineural deafness (Kashtan et al., 2018). The resulting variants have been scored according to the standard 5‐tiered system, based on data from population‐ and disease‐specific databases, type, and segregation of the variant (Richards et al., 2015). Only variants with a score from 3 (uncertain significance) to 5 (pathogenic) are reported (they have been submitted to the ClinVar gene variant database with the accession numbers from SCV001245125.1 to SCV001245153.1) (https://www.ncbi.nlm.nih.gov/clinvar/). The main clinical features of the patients with ATS have been recorded according to the Human Phenotype Ontology (HPO) (http://human‐phenotype‐ontology.github.io/) and archived for analysis into a dedicated database together with the genetic data. Statistical analysis has been carried out with the IBM SPSS Statistics for Windows, version 22 (IBM Corp.), using the chi‐square test for comparisons between groups and the log‐rank test for ascertaining differences in the ESRD rates. The local ethical committee approval has been requested and obtained for this study (Prot. N. 1042/2018).

## 3 RESULTS

**FIGURE 1:**
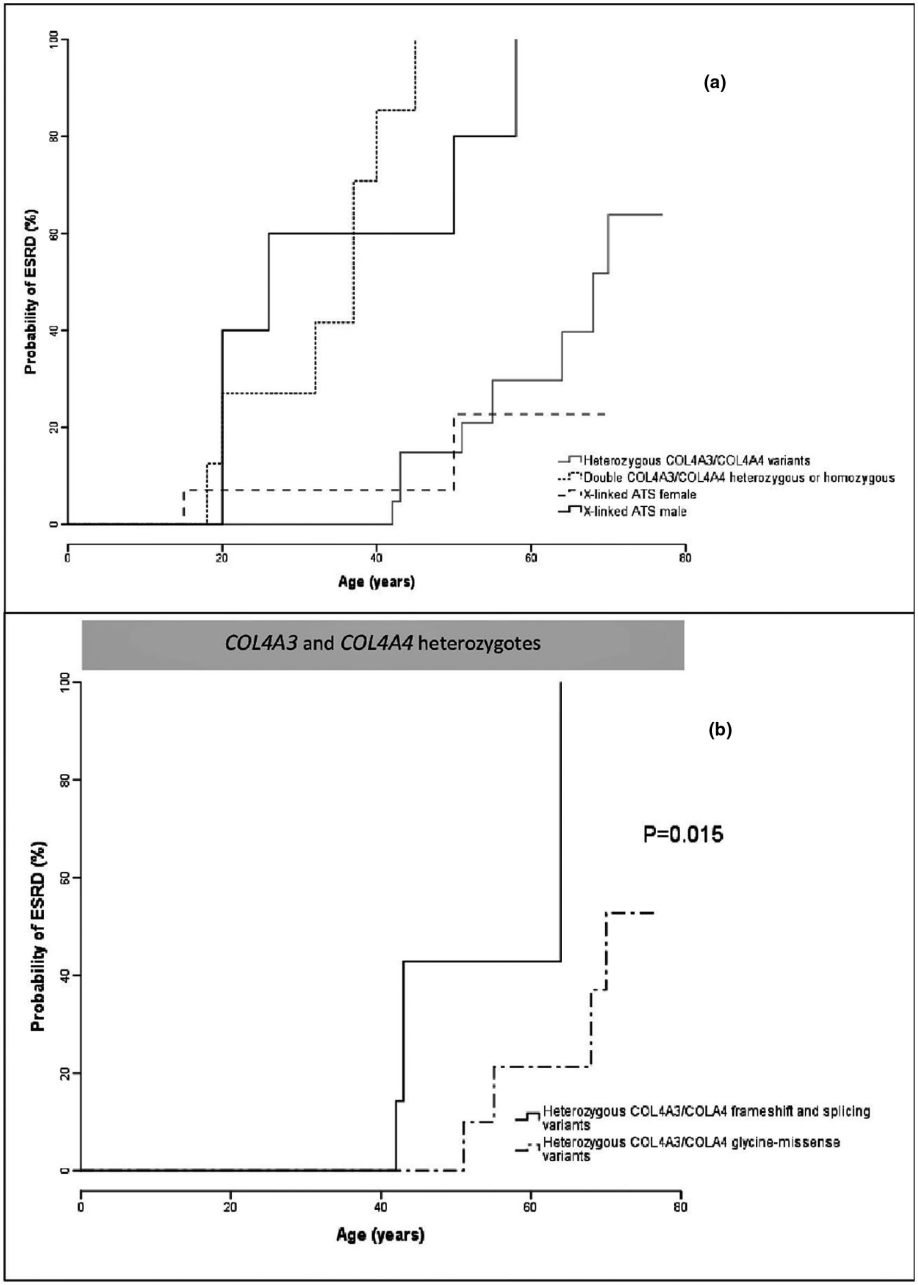
Probability of end‐stage renal disease for the heterozygous *COL4A3*/*COL4A4* ATS patients compared to the recessive and X‐linked counterparts (Panel a) and for the heterozygous *COL4A3*/*COL4A4* heterozygous carriers of truncating variants vs. those with missense substitutions in the glycine domains (Panel b)

Out of 74 probands tested for a clinical suspicion of ATS, 34 showed the presence of a variant in a COL4 gene. Among those, 11 showed variants in *COL4A5*, 12 in *COL4A4*, and 11 in *COL4A3* (Table 1 and Table S1). Considering the type of variant, 47% of them (16/34) were substitutions of glycine amino acid residues in the collagenous domains of the genes, 8/34 (24%) were non‐glycine missense variants, and 29% (10/34) were truncating the COL4 protein synthesis. Clinical and genetic data were examined for a total of 76 probands and affected family members (audiometric, ocular, and electron microscopy bioptic data were available for a minority of them) (Table 1 and Table S1): In the 11 families with *COL4A5* variants, out of the 32 patients (14 males and 18 females) there was an evolution to renal failure in 50% of the males and 11% of the females at a mean age of 35 and 63 years, respectively (Table 1). Autosomal recessive ATS was diagnosed in 10 patients from 7 families (6 compound heterozygotes and 1 homozygous for *COL4A3* and *COL4A4* variants): one third of the patients developed renal failure at a mean age of 33 years, 87% (7 out of 8 for whom data were available) had sensorineural hearing loss, and 75% (3 out of 4) had typical ATS ocular anomalies (Table 1 and Table S1). Heterozygous variants in *COL4A3* and *COL4A4* were found in 14 families for a total of 34 patients: of them, 96% presented with microhematuria and 8 out 34 (24%) developed renal failure at a mean age of 65 years (Table 1). Figure 1, Panel A shows that the probability of ESRD was significantly lower for heterozygous ATS compared to XLS males and recessive ATS (log rank 34.49 and 15.69, respectively; *p* < 0.001 for both), but did not differ from that of XLS females (log rank 0.22, *p* = 0.6). Table 2 stratifies the 34 ATS patients with heterozygous *COL4A3*/*COL4A4* mutations according to the site and type of mutations: of those with substitutions of the glycine amino acid in the collagenous domains, 19% developed ESRD, whereas heterozygotes for loss‐of‐function variants showed a more aggressive disease (66% with ESRD) and a lifetime probability of ESRD significantly higher than for carriers of glycine substitution (log rank 5.86, *p* = 0.015, Figure 1, Panel B). The three patients with *COL4A3*/*COL4A4* non‐glycine heterozygous missense variants were not included into the analysis due to their unknown clinical significance (Table S1).

**TABLE 1:** Phenotypic features of patients with hemizygous and heterozygous *COL4A5* variants, heterozygous, and compound heterozygous (or homozygous) *COL4A3* and *COL4A4* variants (numbers and percentages refer to patients for whom the clinical information was available)

Clinical features	COL4A5		COL4A3 and COL4A4	
	Heterozygotes (*N* = 18)	Hemizygotes (*N* = 14)	Compound heterozygotes or homozygotes (*N* = 10)	Single Heterozygotes (*N* = 34)
Age at evaluation (years and range)	44.6 (7–70)	23.6 (3–58)	33.6 (12–50)	42.8 (6–77)
M/F	0/18	14/0	2/8	23/11
Microhematuria (HP:0002907)	17/17	6/7 (86%)	6/6	27/28 (96%)
Proteinuria (HP:0000093)	4/16 (25%)	5/8 (63%)	4/5 (80%)	10/26 (38%)
Renal failure (HP:0003774)	2/18 (11%)	6/12 (50%)	7/10 (70%)	8/34 (24%)
Mean age of renal failure (years and confidence interval)	62.9 (54.1–71.9)	34.8 (19.1–50.5)	33.0 (25.7–40.3)	65.1 (59.0–71.3)
Glomerular basal membrane anomalies (HP:0012577) (HP:0030034)	3/3	2/2	2/2	3/3
Hypoacusia (HP:0000407)	3/6 (50%)	5/6 (83%)	7/8 (87.5%)	3/7 (43%)
Ocular anomalies (HP:0000488)	1/3 (33%)	1/2 (50%)	3/4 (75%)	0/3

**TABLE 2:** Clinical features of the 34 ATS patients with heterozygous *COL4A3* and *COL4A4* mutations stratified according to the site and type of mutations (numbers and percentages are relative to patients for whom clinical information was available)

Clinical features	COL4A3 and COL4A4		
	Frameshift/splicing (*N* = 10)	Glycine missense (*N* = 21)	Non‐glycine missense (*N* = 3)
Age at evaluation (years and range)	39.8 (13–53)	45.5 (13–77)	33 (6–51)
M/F	7/3	15/6	1/2
Microhematuria (HP:0002907)	5/5 (100%)	19/20 (95%)	3/3 (100%)
Proteinuria (HP:0000093)	0/5	9/18 (50%)	1/3 (33%)
Renal failure (HP:0003774)	4/6 (66%)	4/21 (19%)	0/3
Mean age of renal failure (years and confidence interval)	54.8 (45.8–63.9)	69.4 (63.3–75.5)	—

## 4 DISCUSSION

Genotype/phenotype correlations in our study are consistent with a semidominant pattern of inheritance of the *COL4A3* and *COL4A4* variants and suggest that the renal functional prognosis of patients with *COL4A3*‐ or *COL4A4*‐positive ATS recapitulates the phenotypic pattern of the X‐linked ATS forms, with the outcome of the *COL4A3* and *COL4A4* heterozygotes being very similar to that of XLAS carrier females, whereas the compound *COL4A3* and *COL4A4* heterozygosity reproduces the severe prognosis of XLAS‐affected males (Figure 1, Panel A). Our results for the XLAS males (evolution to ESRD in 75% of the males by the age of 30 years) show a similar prognosis to the largest series published so far, which reports a 76.5% probability of ESRD by the age of 30 years, further subdivided into 90% for the truncating variants, 50% for the missense and 70% for the splice site (Jais et al., 2000). Also for carrier women, our 10% of ESRD by the age of 40 years is very similar to the reported 12% of ESRD by same age described in the largest carrier cohort (Jais et al., 2003). For autosomal recessive ATS, we have registered a median age of ESRD of 37 years in our 10 patients, which is more favorable than the median ESRD onset of 21 years of age reported in a recent systematic review (Lee et al., 2019), but probably suffers from the low number of observations in our study. Finally, for the heterozygous *COL4A3*/*COL4A4* pathogenic variants carriers, our data are consistent with a recent systematic review on *COL4A3*/*COL4A4* nephropathy (Matthaiou et al., 2020), in which the rate of ESRD is 15.1% at a mean age of 52.8 years. In the 34 patients from 14 families with heterozygous *COL4A3* or *COL4A4* variants, we report ESRD in 24% of the subjects (Table 2) at a mean age of 65.1 years. Although it cannot be ruled out that in our 14 heterozygous families we could have overlooked cryptic mutations in the other allele resulting in classification errors, it is however reassuring that only 2 out 14 cases were sporadic, whereas the others were familial and consistent with a *bona fide* autosomal dominant transmission of the phenotype. On the other hand, it must be warned that the present and the previous genetic studies (Matthaiou et al., 2020) probably present an overestimation of the risk, due to a selection bias for the most severe cases, whereas the majority of families with isolated microhematuria or cases with no renal symptoms are not referred to a genetic analysis. By considering the type of heterozygous *COL4A3* and *COL4A4* variants, patients with loss‐of‐function mutations presented a significantly higher risk of developing ESRD than those with missense changes in the glycine residue (Figure 1, panel B), as already known for the *COL4A5* gene (Jais et al., 2000, 2003). Evidences are instead too few for the heterozygous non‐glycine missense variants, which were found in 3 patients and have not been included in the analysis due to their interpretation of unknown clinical significance and their uncertain causality link to the renal disease (Table S1).

In conclusion, the analysis of the *COL4A3*, *COL4A4*, and *COL4A5* genes using NGS technology is a powerful tool for the dissection of the underlying genetic cause of the hematuric nephropathies and a valid help in the diagnosis of disorders arising from abnormalities of the collagen IV α345 molecules (Artuso et al., 2012). The association of an accurate evaluation of patients with a clinical diagnosis of collagen IV‐related GBM nephropathy and the analysis of the three genes in a single step allows the identification of the transmission patterns and the individuation of at‐risk family members, together with the possibility to infer the renal prognosis in subjects with abnormalities of the collagen IV α345 molecules.

## CONFLICT OF INTEREST

The authors have declared no conflicts of interest for this article.

## AUTHORS’ CONTRIBUTIONS

Vera Uliana and Antonio Percesepe conceived and designed the study; Paola Sebastio performed the experiments; Matteo Riva and Francesco Bonatti analyzed the data; Diana Carli, Claudio Ruberto, Laura Bianchi, Claudio Graziano, Irene Capelli, Flavio Faletra, Roberto Pillon, Teresa Mattina, Alberto Sensi performed the clinical characterization of the patients; Vera Uliana and Antonio Percesepe wrote the paper.

## Funding information

This research was supported by the “Fondazione Emma ed Ernesto Rulfo per la Genetica Medica” (Italy).

## Supporting information

Table S1

## Data Availability

Data available on request from the authors.
