# Supplementary material for: Deciphering the pathogenesis of the COL4‐related hematuric nephritis: A genotype/phenotype study
Source: Mol Genet Genomic Med. 2020 Dec 24;9(2):e1576. doi: 10.1002/mgg3.1576 (PMC8077073; doi:10.1002/mgg3.1576)
Supplement: Supplementary file 1 — Table S1 [file MGG3-9-e1576-s001.docx]

**Supplementary Table 1. List of the variants in the COL4A5, COL4A4, COL4A3 genes found in the analysis of the Alport syndrome families and their clinical features**

| **Sex** | **Age** | **Gene** | **Variant** | **Variant**  **Classification*** | **Urinalysis** | **CDK stage** | **Age at ESRD** | **Audiometric evaluation** | **Ophtalmological evaluation** | **GBM anomalies (EM)** |
| --- | --- | --- | --- | --- | --- | --- | --- | --- | --- | --- |
| M | 58, deceased | *COL4A5* | c.3473G>A (p.Gly1158Glu) | 4 | Microhaematuria | End stage | n.a. | SNHL | n.a. | n.a. |
| M | 35 | *COL4A5* | c.385G>A (p.Gly129Arg) | 5 | Microhaematuria | End stage | 26 | SNHL | Myopia | Thick |
| M | n.a. | *COL4A5* | c.385G>A (p.Gly129Arg) | 5 | n.a. | End stage | 20 | n.a. | n.a. | n.a. |
| M | 18 | *COL4A5* | c.4221delA (p.Thr1408Leufs*146) | 4 | Microhaematuria, Proteinuria | no |  | SNHL | n.a. | n.a. |
| M | n.a., deceased | *COL4A5* | c.4221delA (p.Thr1408Leufs*146) | 4 | n.a. | End stage | n.a. | n.a. | n.a. | n.a. |
| M | n.a. | *COL4A5* | c.3403A>G (p.Ile1135Val) | 3 | n.a. | no |  | SNHL | n.a. | n.a. |
| M | n.a. | *COL4A5* | c.3403A>G (p.Ile1135Val) | 3 | Nephrotic syndrome | n.a. |  | n.a. | n.a. | n.a. |
| M | 19 | *COL4A5* | c.1799G>T (p.Gly600Val) | 4 | Microematuria | no |  | Normal | Normal | Thick, split and thin |
| M | 15 | *COL4A5* | c.1799G>T (p.Gly600Val) | 4 | Microematuria | no |  | n.a. | n.a. | n.a. |
| M | 4 | *COL4A5* | c.1120D>A (p.Gly374Arg) | 4 | Microematuria | no |  | n.a. | n.a. | n.a. |
| M | 13 | *COL4A5* | c.865G>A (p.Gly289Ser) | 4 | n.a. | n.a. |  | n.a. | n.a. | n.a. |
| M | n.a. | *COL4A5* | c.4821G>C (p.Met1607Ile) | 3 | n.a. | End stage | 50 | n.a. | n.a. | n.a. |
| M | 3 | *COL4A5* | c.3623G>A (p.Gly1208Glu) | 4 | Microematuria | no |  | n.a. | n.a. | n.a. |
| M | 47 | *COL4A5* | c.3623G>A (p.Gly1208Glu) | 4 | Microematuria | End stage | 20 | SNHL | n.a. | n.a. |
| F | 34 | *COL4A5* | c.3473G>A (p.Gly1158Glu) | 4 | Microematuria | no |  | Normal | n.a. | Thin |
| F | 67 | *COL4A5* | c.385G>A (p.Gly129Arg) | 5 | Microematuria | no |  | n.a. | n.a. | n.a. |
| F | 70 | *COL4A5* | c.3473G>A (p.Gly1146Glu) | 4 | n.a. | End stage | 50 | SNHL | n.a. | n.a. |
| F | 41 | *COL4A5* | c.3473G>A (p.Gly1146Glu) | 4 | Microematuria | no |  | Normal | Retinal flecks | n.a. |
| F | 34 | *COL4A5* | c.4221delA (p.Thr1408Leufs*146) | 4 | Microematuria | no |  | n.a. | n.a. | n.a. |
| F | 60 | *COL4A5* | c.4221delA (p.Thr1408Leufs*146) | 4 | Microematuria | no |  | n.a. | n.a. | n.a. |
| F | 7 | *COL4A5* | c.3403A>G (p.Ile1135Val) | 3 | Microematuria | no |  | n.a. | Normal | n.a. |
| F | 46 | *COL4A5* | c.3403A>G (p.Ile1135Val) | 3 | Microematuria | no |  | n.a. | n.a. | n.a. |
| F | n.a. | *COL4A5* | c.3403A>G (p.Ile1135Val) | 3 | Microematuria | no |  | n.a. | n.a. | n.a. |
| F | 14 | *COL4A5* | c.2692A>G (p.Met898Val) | 5 | Microematuria | no |  | SNHL | n.a. | Thick and thin |
| F | 45 | *COL4A5* | c.1799G>T (p.Gly600Val) | 4 | Microematuria | no |  | n.a. | n.a. | n.a. |
| F | 70 | *COL4A5* | c.1799G>T (p.Gly600Val) | 4 | Microematuria | no |  | n.a. | n.a. | n.a. |
| F | n.a. | *COL4A5* | c.1120D>A (p.Gly374Arg) | 4 | Microematuria | no |  | n.a. | n.a. | n.a. |
| F | 52 | *COL4A5* | c.865G>A (p.Gly289Ser) | 4 | Microematuria | no |  | SNHL | n.a. | n.a. |
| F | 35 | *COL4A5* | c.4821G>C (p.Met1607Ile) | 3 | Microematuria | no |  | n.a. | n.a. | n.a. |
| F | 62 | *COL4A5* | c.4821G>C (p.Met1607Ile) | 3 | Microematuria | no |  | n.a. | n.a. | n.a. |
| F | 25 | *COL4A5* | c.3623G>A (p.Gly1208Glu) | 4 | Microematuria | no |  | Normale | Normal | Thin |
| F | 52, deceased | *COL4A5* | c.3623G>A (p.Gly1208Glu) | 4 | Microematuria | End stage | 15 | n.a. | n.a. | n.a. |
| F | 48 | *COL4A4* | c.755G>A (p.Gly252Asp) | 4 | Microematuria | End stage | 37 | SNHL | Unilateral optic atrophy | n.a. |
|  |  | *COL4A4* | c.755G>A (p.Gly252Asp) | 4 |  |  |  |  |  |  |
| M | n.a. | *COL4A4* | c.755G>A (p.Gly252Asp) | 4 | n.a. | End stage | 37 | n.a. | n.a. | n.a. |
|  |  | *COL4A4* | c.755G>A (p.Gly252Asp) | 4 | n.a. |  |  | n.a. | n.a. | n.a. |
| F | n.a. | *COL4A4* | c.755G>A (p.Gly252Asp) | 4 |  | End stage | 32 |  |  |  |
|  |  | *COL4A4* | c.755G>A (p.Gly252Asp) | 4 |  |  |  |  |  |  |
| M | 34 | *COL4A4* | c.3581T>C (p.Leu1194Ser) | 3 | n.a. | End stage | 20 | SNHL | Bilateral cataract | n.a. |
|  |  | *COL4A4* | c.2717-5A>T (p.?) | 4 |  |  |  |  |  |  |
| F | 16 | *COL4A4* | c.2630G>A (p.Arg877Gln) | 3 | Microematuria | NO |  | SNHL | n.a. | n.a. |
|  |  | *COL4A4* | c.680G>A (p.Arg227His) | 3 |  |  |  |  |  |  |
| F | 18 | *COL4A3* | c.2488+2delGGT (p.?) | 4 | Microematuria | NO |  | SNHL | Lenticonus | n.a. |
|  |  | *COL4A3* | c.2620delG (p.Gly874Aspfs*9) | 4 |  |  |  |  |  |  |
| F | 12 | *COL4A4* | c.2940dupT (p.Gly981Trp*9) | 4 | Microematuria | NO |  | Normale | Normal | Thin GBM |
|  |  | *COL4A4* | c.1820C>T (p.Ala607Val) | 3 |  |  |  |  |  |  |
| F | 47 | *COL4A3* | c.343G>A (p.Gly115Arg) | 4 | Microematuria | End stage | 45 | SNHL | n.a. | n.a. |
|  |  | *COL4A3* | c.361G>A (p.Gly121Ser) | 4 |  |  |  |  |  |  |
| F | 50 | *COL4A3* | c.343G>A (p.Gly115Arg) | 4 | n.a. | End stage | 40 | SNHL | n.a. | n.a. |
|  |  | *COL4A3* | c.361G>A (p.Gly121Ser) | 4 |  |  |  |  |  |  |
| F | 44 | *COL4A3* | c.2489-1G>A (p.?) | 4 | Microematuria | End stage | 18 | SNHL | n.a. | Thick, split and thin |
|  |  | *COL4A3* | c.2489-1G>A (p.?) | 4 |  |  |  |  |  |  |
| M | 6 | *COL4A3* | c.1084C>T (Pro362Ser) | 3 | Microematuria | NO |  | Normal | n.a. | n.a. |
| F | 42 | *COL4A3* | c.1084C>T (Pro362Ser) | 3 | Microematuria | NO |  | n.a. | n.a. | n.a. |
| F | 13 | *COL4A3* | c.1855G>A (p.Gly619Arg) | 5 | Microematuria | NO |  | n.a. | n.a. | n.a. |
| M | 13 | *COL4A4* | c.1194_1195AinsAGGAA (p.Ala399Argfs*7) | 4 | Microematuria | NO |  | n.a. | n.a. | n.a. |
| M | 69 | *COL4A4* | c.2717-5A>T (p.?) | 4 | n.a. | End stage | 64 | SNHL | n.a. | n.a. |
| M | n.a. | *COL4A4* | c.2717-5A>T (p.?) | 4 | n.a. | End stage |  | n.a. | n.a. | n.a. |
| M | 53 | *COL4A4* | c.4522+4T>G (p.?) | 4 | Microematuria | NO |  | SNHL | n.a. | n.a. |
| F | 51 | *COL4A4* | c.1820C>T (p.Ala607Val) | 3 | Microematuria | NO |  | n.a. | n.a. | n.a. |
| M | 45 | *COL4A4* | c.2940dupT (p.Gly981Trp*9) | 4 | Microematuria | NO |  | n.a. | n.a. | n.a. |
| M | 28 | *COL4A4* | c.1580delG (p.Gly527Valfs*126) | 4 | Microematuria | NO |  | n.a. | n.a. | n.a. |
| F | 42, deceased | *COL4A4* | c.1580delG (p.Gly527Valfs*126) | 4 | n.a. | End stage |  | n.a. | n.a. | n.a. |
| F | 43, deceased | *COL4A4* | c.1580delG (p.Gly527Valfs*126) | 4 | n.a. | End stage |  | n.a. | n.a. | n.a. |
| F | 53 | *COL4A4* | c.1580delG (p.Gly527Valfs*126) | 4 | n.a. | NO |  | n.a. | n.a. | n.a. |
| M | 14 | *COL4A4* | c.3044G>A (p.Gly1015Glu) | 4 | Microematuria | NO |  | Normal | Normal | n.a. |
| M | 52 | *COL4A4* | c.3044G>A (p.Gly1015Glu) | 4 | Microematuria | NO |  | n.a. | n.a. | n.a. |
| M | n.a | *COL4A4* | c.3044G>A (p.Gly1015Glu) | 4 | Microematuria | NO |  | n.a. | n.a. | n.a. |
| M | 18 | *COL4A3* | c.4045G>A (p.Gly1349Ser) | 4 | Microematuria | NO |  | Normal | Normal | n.a. |
| F | 50 | *COL4A3* | c.4045G>A (p.Gly1349Ser) | 4 | Microematuria | NO |  | n.a. | n.a. | n.a. |
| F | n.a. | *COL4A3* | c.4045G>A (p.Gly1349Ser) | 4 | Microematuria | NO |  | n.a. | n.a. | n.a. |
| M | n.a. | *COL4A3* | c.4045G>A (p.Gly1349Ser) | 4 | Microematuria | NO |  | n.a. | n.a. | n.a. |
| M | 52 | *COL4A3* | c.2207G>T (p.Gly736Val) | 4 | Microematuria | Stage 3 | 51 | n.a. | n.a. | Thick and Thin |
| M | 16 | *COL4A3* | c.2207G>T (p.Gly736Val) | 4 | Microematuria | NO |  | n.a. | n.a. | n.a. |
| F | 14 | *COL4A3* | c.2207G>T (p.Gly736Val) | 4 | Microematuria | NO |  | n.a. | n.a. | n.a. |
| M | 35 | *COL4A3* | c.2207G>T (p.Gly736Val) | 4 | Microematuria | NO |  | n.a. | n.a. | n.a. |
| M | 64, deceased | *COL4A3* | c.2207G>T (p.Gly736Val) | 4 | Microematuria | NO |  | n.a. | n.a. | n.a. |
| F | 62 | *COL4A4* | c.836G>A (p.Gly279Glu) | 4 | Microematuria | Stage 3 |  | n.a. | n.a. | Thin |
| M | 35 | *COL4A4* | c.836G>A (p.Gly279Glu) | 4 | Normal | NO |  | n.a. | n.a. | n.a. |
| F | 38 | *COL4A4* | c.836G>A (p.Gly279Glu) | 4 | Microematuria | NO |  | n.a. | n.a. | n.a. |
| M | 70 | *COL4A4* | c.836G>A (p.Gly279Glu) | 4 | Microematuria | End stage | 68 | n.a. | n.a. | n.a. |
| M | 70 | *COL4A4* | c.836G>A (p.Gly279Glu) | 4 | n.a. | End stage |  | n.a. | n.a. | n.a. |
| M | 55 | *COL4A3* | c.4235G>T (p.Gly1412 Val) | 5 | Microematuria | NO |  | SNHL | n.a.a | Thin and split |
| M | 32 | *COL4A3* | c.4235G>T (p.Gly1412 Val) | 5 | Microematuria | NO |  | Normal | Normal | n.a. |
| M | 77, deceased | *COL4A3* | c.4235G>T (p.Gly1412 Val) | 5 | Microematuria | NO |  | n.a. | n.a. | n.a. |
| M | 13 | *COL4A4* | c.2489-1G>A (p.?) | 4 | Microematuria | NO |  | n.a. | n.a. | n.a. |

***The variant classification has been performed according to the standard 5-tiered scoring system of the American College of Medical Genetics and Genomics, including (1) benign, (2) likely benign, (3) uncertain significance, (4) likely pathogenic, (5) pathogenic. Only variants from 3 to 5 are considered.**

**SNHL= Sensoneurinal hearing loss, EM = Electron microscopy, CDK= Chronic Disease kidney, ESRD = End Stage Renal Disease, n.a = not available**
